# Automated Breast Volume Assessment Derived From Digital Breast Tomosynthesis Images Compared to Mastectomy Specimen Weight and Its Applications in Cosmetic Optimisation

**DOI:** 10.7759/cureus.19642

**Published:** 2021-11-16

**Authors:** Asma Munir, Anita M Huws, Saira Khawaja, Sohail Khan, Simon Holt, Yousef Sharaiha

**Affiliations:** 1 Breast Surgery, Prince Philip Hospital, Llanelli, GBR

**Keywords:** digital breast tomosynthesis (dbt), breast cancer, mastectomy, volume estimation, reconstruction

## Abstract

Background: Estimating the size and volume of the breast preoperatively is an important step in surgical planning for many breast procedures such as immediate implant-based breast reconstructions and reduction mammoplasties. Breast volume estimation helps in appropriate implant selection preoperatively.

Objectives: The aim of this study was to objectively evaluate the estimation of breast weight by automatic volumetric breast assessment in digital breast tomosynthesis (DBT) using Quantra™ 2.2 Breast Density Assessment Software (Hologic Inc., Marlborough, Massachusetts, United States). Methods: Breast specimen weight after mastectomy and volume estimated by Quantra software were recorded.

Results: Volume assessment obtained from Quantra software showed a high correlation with actual mastectomy specimen weight, with Pearson’s correlation coefficients of 0.952.

Conclusions: The automated DBT-derived breast volume using the Quantra software is a simple and practical method to assess breast size and weight preoperatively.

## Introduction

Estimating the size and volume of the breast preoperatively is an important step in surgical planning for many breast procedures such as immediate implant-based breast reconstructions and reduction mammoplasties [1]. Breast volume estimation helps in appropriate implant selection preoperatively. In clinical practice, adequate preoperative evaluation usually depends on the experience, skill, and ability of the surgeon. A number of methods have been described in the literature for the assessment of breast volume to enhance clinical evaluation [1-3]. However, there is no consensus on the best approach. Anthropomorphic measurements are reliable, cheap, fast, and reproducible, but the validity and application of these different derived formulas are debatable. Studies using virtual assessment systems like Crisalix® (Crisalix SA, Lausanne, Switzerland).and VECTRA XT® (Canfield Scientific Inc., Bielefeld, United States) have shown accurate prediction of breast volume to guide an implant size [4-6]. However, they are costly and require extra hospital visits for patients. Automated breast volume estimation has previously been described by Gubern-Merida using Volpara™ software (Volpara Health Technologies Ltd, Wellington, New Zealand) [7]. Unfortunately, often this data is not utilized in clinical practice. 

In this study, we describe the use of the Quantra™ 2.2 Breast Density Assessment Software (Hologic Inc., Marlborough, Massachusetts, United States) to predict resected breast volume/weight. This software is designed to assess breast density and volume using diagnostic digital breast tomosynthesis (DBT) images. This information can help the surgeon select the appropriate size of the implant for immediate breast reconstruction. 

## Materials and methods

This was a retrospective review of non-consecutive patients who underwent a mastectomy in the Prince Philip Hospital breast unit between January 2016 and December 2019. Patients were excluded if the Quantra software data was not available or the mastectomy specimen weight was not recorded at the time of surgery. Patients were also excluded if they had undergone a previous augmentation or an implant-based reconstruction. 

During the study period, a total of 1060 women with breast cancer were diagnosed and treated at the Prince Philip Hospital. Of these, 301 (28.4%) patients underwent mastectomies and 117 patients were diagnosed via screening programme and did not have Quantra data available; they were excluded. Another 47 patients were excluded where data for breast weight was not available. Hence, 137 patients were included in the study.

Patient demographics, tumour characteristics, Quantra-derived automated volume estimation and breast specimen weight were recorded. If the patient had undergone a wide local excision and required mastectomy for positive margins, the weight of wide local excision was added to the mastectomy weight. A 1:1 conversion of volume to weight was used for this comparison (1g =1cm3) [8]. In the subgroup of patients in which an immediate implant-based reconstruction was carried out, the implant style and volume selected was also recorded.

The Quantra software is a machine-learning algorithm that analyses each patient's breast tissue pattern and texture to provide an unbiased breast density assessment [9]. The acquisition system in the Digital Imaging and Communications in Medicine (DICOM) header of the Hologic DBT software estimates the amount of fibro-glandular tissue that an x-ray must have penetrated in order to deposit a measured amount of energy at the detector. The amount of fibro-glandular tissue at each pixel level is then reported in centimetres. These pixel-by-pixel based values are aggregated to give the volume of fibro-glandular tissue, in cubic centimetres. Breast density is calculated as a ratio of fibro-glandular tissue and total breast volume estimated.

Data were analysed on SPSS Statistics for Windows, Version 19 (Released 2010. IBM Corp., Armonk, N, United States). Data for normally distributed variables were expressed as mean and standard deviation and medians and interquartile range for non-normally distributed variables. Correlation was evaluated between the breast volume as assessed by the Quantra software and the actual mastectomy specimen weight. This was also applied between the breast volume assessed by Quantra and the actual implant size used. For both subsets, the strength of linear relationships was evaluated using Pearson’s correlation coefficient. A value of p < 0.05 was considered statistically significant.

## Results

During the study period, a total of 1060 women with breast cancer were diagnosed and treated at Prince Philip Hospital. Of these 301 patients underwent mastectomies and 117 patients were diagnosed via screening programme and did not have Quantra data available. Breast weight data was lacking for another 47 patients. Hence, 137 patients were included in the study. Seven of the patients underwent bilateral mastectomies and 17 patients out of the 137 had immediate implant-based breast reconstruction. 

The median age of the cohort was 66 years (23-90 years). The mean mastectomy weight was 841gm (117 - 3084gm).

**Figure 1:**
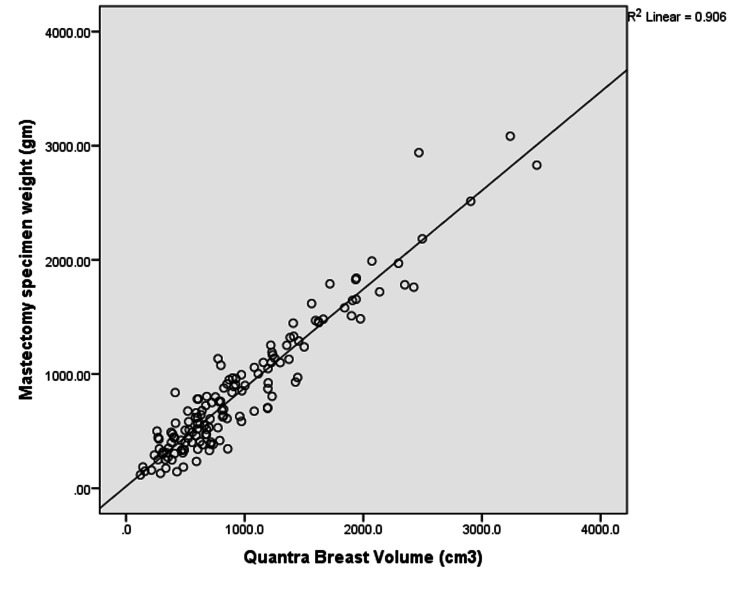
Comparison of breast volume obtained from Quantra™ software and mastectomy specimen weight (N=144) x axis: Quantra Breast Volume (cm3); y axis: Mastectomy Specimen Weight (gm)

**Figure 2:**
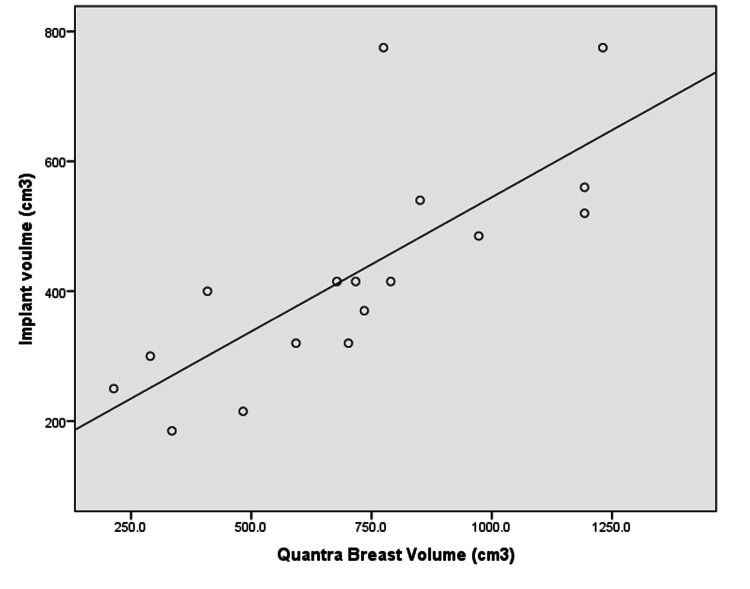
Comparison of breast volume obtained from Quantra™ software and Implant volume for patients who underwent immediate implant-based reconstructions (N=17) x-axis: Quantra Breast Volume (cm3); y-axis:Implant Volume (cm3)

There was a strong positive correlation between the breast volume estimated by Quantra software and mastectomy weight. The correlation coefficient was 0.952 and p-value was < 0.0001 (Figure 1). For the subgroup of 17 patients who underwent immediate implant based reconstructions, the correlation between the breast volume estimated by Quantra software and implant size was modest (r=0.758 and p = 0.001) (Figure 2).

## Discussion

In the medical literature, many different methods have been described to assess breast volume preoperatively. However, no one technique has been universally adopted because of variable reliability. Many of these methods are difficult to reproduce, impractical in clinical settings and often not cost-effective [1,2,10].

Techniques described in the past are diverse. Smith described making a plaster cast of the chest and measuring the amount of sand required to fill it [11]. Morris used a large syringe to act as a portable mammometer [12]. The Grossman-Rounder device [13] utilized a variable cone, which can overestimate the volume in small or firm breasts if they do not fill the apex. Bouman suggested a water displacement method; however, this may underestimate the volume as it may not adequately measure the tissue lateral to the pectoral fold [14]. All these methods are impractical in the clinical setting as they require special instruments and can be inconvenient to both the patient and the surgeon [1,2]. Many different formulas have also been described in the literature based on anthropometric measurements such as Westreich’s formula for the ‘ideal’ breast volume based on manubrium-to-nipple and the nipple-to-nipple distance [15], and by Otiefy, based on the measurements of breast circumference [16]. The former method is complicated limiting its practical use, whereas the latter formula is based on a single measurement, ignoring many other variables important in estimating breast volume (projection, ptosis etc.) [17]. Mathematical derived formulas based on the measurement of 2D mammographic images ignore the fact that compression has been applied when acquiring these images [18]. 

Other methods used effectively for breast volume estimation utilize three-dimensional imaging such as MRI [19,20], three-dimensional topographic imaging, [21-22] and simulation software using computer-assisted custom-made implants [23-25]. However, all these methods are expensive requiring additional imaging, clinical time and patient visits. The current cost of Quantra software licence is a one-off payment of £17,500. Once installed, the service cost is covered by the service on the mammogram. On the other hand, Vectra XT costs USD 40,000 (nearly £29,000) and Crisalix requires a continuous subscription costing nearly £4,000 or more per year [26]. The cost of 3D CT and MRI scans vary across different places and in the United Kingdom (UK) they cost approximately £250 and £500 per patient, respectively [27]. In National Health Services (NHS), the main issue is adding these costs to the treatment and the logistics of doing these investigations when actually they can be avoided if a DBT is already available and has been done to establish the diagnosis.

Previously reported mammographic estimates of breast volume using the Volpara software has shown variable results with Gubern-Mérida et al [7] showing excellent correlation with MRI based breast volume estimation (r= 0.97) but a UK based study was able to show only a modest prediction the weight in 73% of the mastectomy specimens [26].

In this study, we have shown that automated breast volume estimation by the Quantra software correlates very well with the operative breast weight. This has not been previously described in the literature. The estimation of breast volume is automatically done by the software while acquiring images for the DBT. This can be included in the clinical assessment of the patient preoperatively. It can then aid in the selection of implant size for immediate reconstruction.

In our study, the correlation between automated breast volume estimation by the Quantra software and implant size was modest. The small number of patients and retrospective nature of this study precluded a detailed analysis, as many other factors important for consideration for implant selection were not recorded, such as patient’s desire to have enlarged breast or need for simultaneous reduction in volume and overall cosmetic satisfaction.

The Quantra automated technique of breast volume estimation is objective, easily accessible and reproducible. Volume estimation is important in achieving the best cosmetic result but the surgeon also needs to consider other significant variables such as natural variations in breast shape, chest wall size (footprint), conus, and ptosis [27]. In addition, preoperative breast volume assessment can be helpful in achieving optimal cosmetic results in therapeutic mammaplasty, reduction mammaplasty, and in guiding the maximum resection volume reasonable in wide excisions.

The limitation of this study is the small sample size, especially with regards to the reconstruction cohort. During the study period, the implant-based reconstruction rate in our unit was 20%; however, most of these patients were excluded from the present study as they were diagnosed through the screening programme and did not have Quantra data available. Moreover, the availability of DBT and the Hologic automated volume and density software (Quantra) is limited at all centres across the UK. At present, in the NHS Breast Screening Programme, initial imaging is by 2D mammography with DBT used only in subsequent assessment. Moreover, the Quantra software is an additional feature that adds to the cost of the machine. 

## Conclusions

The automated DBT-derived breast volume using the Quantra software is a simple and practical method to assess breast size and weight preoperatively. The estimation can be used to aid clinical decision making for preoperative breast volume estimation and implant size calculation.
